# A Combined Approach for the Characterization of Small Ruminant Lentivirus Strains Circulating in the Islands and Mainland of Greece

**DOI:** 10.3390/ani14071119

**Published:** 2024-04-06

**Authors:** Ilias Bouzalas, Evangelia D. Apostolidi, Daniela Scalas, Evangelia Davidopoulou, Taxiarchis Chassalevris, Sergio Rosati, Barbara Colitti

**Affiliations:** 1 Hellenic Agricultural Organization—DEMETER, Veterinary Research Institute, Campus of Thermi, 57001 Thessaloniki, Greece; bouzalas@elgo.gr (I.B.); eviapostolidi@hotmail.com (E.D.A.); taxiarchis@hotmail.com (T.C.); 2 Department of Veterinary Sciences, University of Turin, L. Braccini 2, 10095 Torino, Italy; daniela.scalas@unito.it (D.S.); sergio.rosati@unito.it (S.R.); 3 AnimalLab, 57001 Thermi, Greece; animallab@yahoo.com

**Keywords:** small ruminant lentiviruses, genetic characterization, next-generation sequencing, serotyping

## Abstract

**Simple Summary:**

Small ruminant lentiviruses are a group of viruses characterized by a high genetic and antigenic variability. In the absence of control measures, they are characterized by high prevalence in goat and sheep farms. In Greece, a country characterized by one of the highest small ruminant populations in Europe, SRLVs are well-known pathogens, but so far, few studies have investigated the circulating viral strains and their prevalence in the islands and mainland. The results of this research highlighted the presence of both A and B subtypes as well as the predominance of a novel A viral cluster. In many cases, the combined serological–molecular approach allowed us to identify more than one viral subtype in a single farm.

**Abstract:**

Small ruminant lentiviruses are a group of viruses infecting goat and sheep worldwide. These viruses exhibit an extraordinary degree of genetic and antigenic variability that severely influence in vivo and in vitro features, as well as diagnostic test results. Small ruminant farming is the most important animal farming business in Greece, with a high impact on the Greek primary economy. Although SRLV infection and its impact on animal production are well established in the country, little is known about the circulating SRLV strains and their prevalence. The aim of this study was to characterize SRLVs circulating in Greece with a combined serological and molecular approach, using the bulk milk matrix collected from 60 farms in different municipalities. This study allowed us to estimate a seroprevalence of around 52% at the herd level. The B1, B2 and A3 subtypes and a novel A viral cluster were identified. Moreover, the amplicon sequencing method allowed us to identify more than one viral subtype in a sample. These results again confirm the high variability of these viruses and highlight the importance of the constant monitoring of viral evolution, in particular in antigens of diagnostic interest.

## 1. Introduction

Small ruminant lentiviruses (SRLV) are a group of viruses that cause chronic multisystemic infections with a high economic impact worldwide [1].

Initially believed to be two distinct viral species, Maedi–Visna Virus (MVV) and Caprine Arthritis Encephalitis Virus (CAEV) are now considered to be a viral continuum and are referred to as one group without host restrictions [2,3].

They have been classified into four main genotypes (A, B, C and E) and several subtypes, based on the sequence analysis of two genomic fragments: the gag–pol (1.8 kb) and the pol (1.2 kb) regions [3].

SRLVs are characterized by a high genetic and antigenic variability, having one of the fastest-evolving genomes thanks to the lack of proofreading ability of their reverse transcriptase [4]. This high heterogeneity strongly influences both the in vivo and in vitro properties of SRLVs, as well as their diagnostic test results [1,5,6,7,8,9,10,11,12,13,14,15,16,17,18,19].

Since their first occurrence in the mid-1990s, SRLVs have spread across Europe, reaching a high prevalence and endemicity in almost the whole continent, likely due to the high density of the small ruminant population together with the intensive management [5] typical of many countries. In a recent publication, de Miguel and colleagues stated that the highest individual SRLV prevalence in Europe for sheep has been reported in Lebanon, Greece and Spain [5]. Although in Greece SRLVs have been well-known pathogens since 1967 [6] and Greece is one of the main goat and sheep farming centers in Europe [7], information regarding the viral strains circulating in this country is limited [6,8,9]. Moreover, in Greece, the small ruminant population has evolved in a wide range of ecological niches that are well adapted to the local environment [10]. A great number of different indigenous sheep and goat breeds can be found on the mainland and islands, representing an important source for studying SRLV evolution.

In addition, small ruminant populations on islands are considered quite isolated from external pathogens due to the implementation of strict controls during transportation, and thus isolated SRLV strains from those animal groups might expand our knowledge on the epidemiology of the virus.

In the absence of effective vaccines, the control of the disease mainly relies on control programs based on the early detection of infection. Thus, compulsory or volunteer control programs have been implemented throughout Europe. In this context, good diagnostic tests are crucial tools for the monitoring and control of SRLV infection. However, the genetic and antigenic variability of SRLVs pose an issue for developing a unique assay able to detect all the possible viral variants, and a gold-standard test is not available to date [11]. Several tests have been applied so far for the diagnosis of SRLV [12,13]. Agar gel immunodiffusion (AGID) and enzyme-linked immunosorbent assay (ELISA) are the most commonly used and prescribed by the WOAH [14]. Of these, ELISA tests are widely used, since their good performance is combined with scalability and cost-effectiveness, while AGID is usually applied to confirm ELISA results [15,16]. However, indirect diagnosis can be affected by the variability of circulating strains, delayed seroconversion, and variations in antibody responses [1,17]. In recent years, several molecular methods have been developed and routinely used for the diagnosis of SRLV infection. PCR represents a valuable tool since it allows for the early detection of viral RNA in different matrices, such as blood, colostrum, milk, semen and several tissues [1,18,19]. Of these, milk can be a valuable matrix for both serological and molecular applications, since it is easily obtained by non-invasive procedures and reflects one of the major routes of transmission [20,21,22,23]. However, low viral loads in latently infected animals and the high viral genetic heterogeneity strongly affect the sensitivity of molecular assays. For these reasons, a combination of serological and molecular assays is often suggested [22,24]. In this context, recent advantages in sequencing techniques represent a good opportunity to overcome some diagnostic issues. Next-generation sequencing technologies (NGS) have been successfully applied to the genetic characterization of SRLVs circulating in different areas [25,26,27].

Therefore, in the present study, a serological and molecular characterization of SRLV strains circulating in the islands and mainland of Greece was carried out using a serological–molecular combined approach and novel sequencing tools.

## 2. Materials and Methods

### 2.1. Sample Selection and Serological Analysis

**Figure 1:**
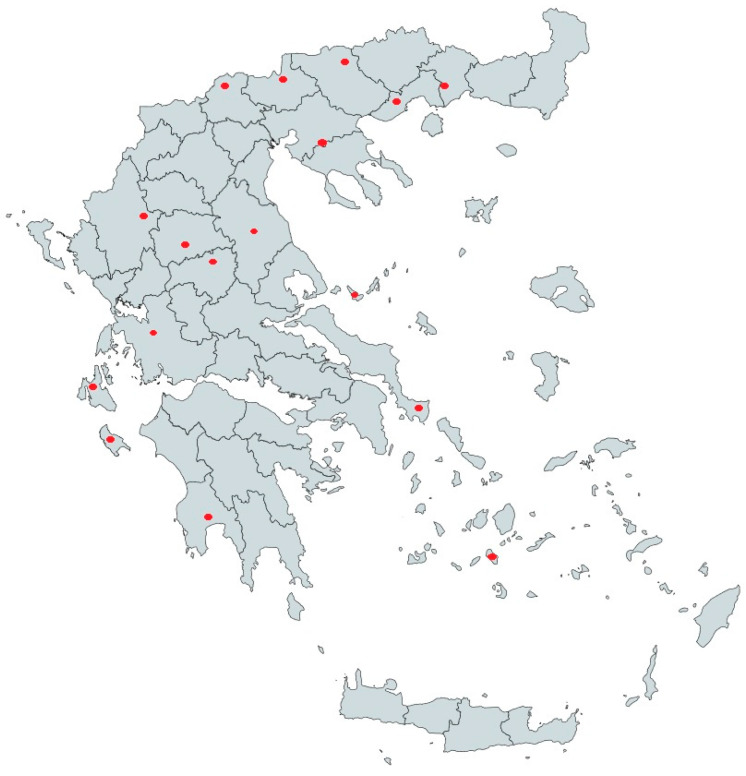
Map of Greece showing regions sampled during the study.

A total of 60 fresh raw bulk milk samples (from 33 goat farms, 21 sheep farms and 6 mixed farms) were collected from different geographical municipalities of Greece (Figure 1). Bulk milk samples belonged to farms without clinical signs of the disease and breed was not a selection criterion for sampling, since most of the enrolled animals exhibited morphological phenotypes of indigenous breeds and crosses of them with foreign ones. The total number of animals that contributed to the tested bulk milk samples were 7446 goats, 2687 sheep and 2444 animals of both species belonging to mixed farms. The demographic distribution of those animals was as follows: 19 herds counting 1666 goats, 10 flocks counting 885 sheep and 2 mixed farms counting 434 animals were kept in islandic regions of Greece, whereas 14 herds counting 5780 goats, 11 flocks counting 1802 sheep and 4 farms mixed counting 2010 animals were kept in the mainland of Greece (Table 1).

Initially, the collected raw bulk milk samples were tested for the presence of antibodies against SRLV using the In3diagnostic SRLV platform (In3Diagnostic, Torino, Italy) [23].

Briefly, 200 ul of defatted milk samples was antibody-tested with the Eradikit™ SRLV screening test (In3Diagnostic, Italy) and positive and doubt samples were serotyped with the Eradikit SRLV genotyping kit (In3Diagnostic, Italy) in which genotype-specific antigens allow discrimination between the A, B and E genotypes. For the screening ELISA, results and sample-to-positive (S/P) ratios were calculated according to the manufacturer’s instructions. For the genotyping ELISA, results were given as indeterminate or positive for 1 (A, B, E) or > 1 (e.g., AB) genotype.

Moreover, 3 additional tissue samples (from sheep lungs) belonging to animals with clinical signs of SRLV infections were also collected from the slaughterhouse and were enrolled in our study as positive controls. These clinically affected animals were derived from three different farms. Sample details are listed in Table 1.

### 2.2. DNA Extraction and SRLV Proviral Amplification

Genomic DNA was extracted from tissues and bulk tank milk samples as previously reported [22], with minor modifications. Briefly, milk somatic cells were concentrated by centrifugation from 40 mL of raw milk at 4000× *g* 20 min at 4 °C, washed twice with PBS 1× and resuspended in 200 μL to obtain the cell pellet, while 50 mg of tissue samples was homogenized and resuspended in 250 μL 1% phosphate-buffered saline (PBS). Proviral SRLV DNA was extracted with DNeasy Blood and Tissue Kit (Qiagen, Hilden, Germany) following manufacturer instructions and quantified with Nanodrop 2000 Spectrophotometer (Thermo Fisher Scientific, Waltham, MA, USA). A previously published nested PCR [28] was used to amplify an approximately 800 bp long sequence of the SRLV *gag–pol* fragment.

Briefly, the first hemi-nested PCR used degenerate primers to amplify a 1.3 kb sequence of the *gag–pol* genes while the second nested PCR was designed to amplify a 0.8 kb sequence of the *gag* gene. PCR products were visualized in 1.5% agarose gel electrophoresis, and positive samples, showing the band of the expected size, were purified using a Gel and PCR purification kit (Macherey-Nagel, Duren, Germany) and were submitted to an external laboratory for Sanger sequencing (BMR Genomics, Padova, Italy). Primer sequences are provided in Supplementary Table S1.

### 2.3. Amplicon Sequencing

Samples that gave discordant results between serology and PCR or with multiple or not clearly defined bands on the agarose gel, or with overlapping peaks in the Sanger chromatograms, were submitted to NGS amplicon sequencing as follows.

Briefly, even in the absence of clear bands on the agarose gel, the first and second nested PCR reactions were mixed and purified using a Gel and PCR purification kit (Macherey-Nagel, Duren, Germany). PCR-amplified DNA was fluorimetrically quantified with a Qubit double-strand DNA (dsDNA) High-Sensitivity assay kit on a Qubit 3.0 instrument (Thermo Fisher Scientific, Waltham, MA, USA) and subjected to tagmentation, amplification and indexing using the Illumina Nextera XT Library prep Kit (Illumina, San Diego, CA, USA) according to the manufacturer’s protocol. Libraries were then diluted to 4 nM concentration, pooled and denatured with 0.2 N sodium acetate. The 12.5 pM paired-end library was spiked with 5% PhiX control and sequenced on an Illumina Miseq platform using a V3–600 cycle chemistry kit.

### 2.4. Sequence Analysis

Sanger sequencing and NGS data were analyzed using Geneious Prime v. 2023.2.1 software as previously reported [25,26] with some modifications.

Briefly, Illumina raw reads were filtered for low quality using FastQC v. 0.12.0 [29]. Qualified reads were then aligned to a batch of reference genomes retrieved from the GenBank database to identify and confirm the viral genotype. The reads were further aligned to the consensus sequence obtained after the first mapping step in order to confirm the genome sequence and to check for the presence of more than one viral genome in the sample, considering a cut-off value of 98% of sequence identity in order to select two distinct viral sequences. Moreover, variant distribution was evaluated for each strain considering the intra-sample Single-Nucleotide Variants (SNPs) relative to the final alignment in Geneious Prime using default parameters (Maximum Variant *p*-value of 10^−6^, Minimum Strand Bias *p*-value of 10^−5^ when exceeding 65% bias) and a conservative frequency of 5% and > 100 reads to avoid sequencing errors and mis-mapping when calling SNPs.

A DeNovo assembly was also performed by Velvet software ver. 1.2.10 [30] and the obtained contigs were compared to the consensus sequence derived from resequencing.

The phylogenetic analysis was based on the partial gag–pol gene fragment, sequenced from proviral DNA, as described by Shah [3]. The 780 bp partial gag fragment of 45 Greek sequences (16 obtained with Sanger and 29 with NGS sequencing) was aligned with 93 reference strains retrieved from GenBank using MUSCLE [31] included within the software Geneious Prime ver. 2023.2.1.

The phylogenetic relationships between the newly characterized and the reference strains was reconstructed using two approaches: the phylogenetic tree was first obtained with a Bayesian method implemented in MrBayes package [32] with the GTR+G+I substitution model (bootstrap values of 1000 replicates), and then confirmed with the maximum likelihood inference method using the Tamura–Nei model (bootstrap values of 1000 replicates) implemented in MEGA11 software [33].

Nucleotide sequences were deposited in GenBank (accession numbers from PP484895 to PP484939).

## 3. Results

### 3.1. Serology

In total, 31 bulk milk samples out of 60 (12 goat, 14 sheep and 5 mixed farms) gave positive results in the screening ELISA test and 18 samples were correctly serotyped. For three samples (three goat farms), a doubtful ELISA result was recorded. Interestingly, among 31 screening-positive samples, 15 gave inconclusive results in the genotyping ELISA test (Table 2).

Considering these findings, we checked each sequence for mismatches in the region encoding the immunodominant capsid epitope employed in serotyping tests. The amino acid alignment showed the presence of mismatches in several samples, as shown in Table 3. For example, samples G002, G021, G024 and G044 that showed weak or negative results in the genotyping ELISA harbored a mismatch in the first amino acid of the capsid epitope, while samples G047 and G048 gave weak and inconclusive results in both the screening and genotyping ELISA and harbored mismatches in their sequences (Figure 1).

The sample size allowed us to estimate a herd-level prevalence of antibody-positive bulk milk samples in Greece of around 52%. Briefly, seropositivity was detected in 12 out of the 31 examined islandic farms (38.7%), whereas higher seropositivity (65.5%) was detected in the farms on the mainland (19 out of the 29 examined farms).

### 3.2. Sequence Analysis

Genomic DNA was extracted from the sixty milk cell pellet samples with an average concentration of 94.26 ng/μL (95% CI, 74.29–114.29), and from three tissue samples with an average concentration of 13.2 ng/μL (95% CI, 6.78–19.62), and underwent the *gag-800* nested PCR. Twenty-four out of thirty-one screening-positive samples showed a band of the expected size in the gag–pol nested PCR. However, only 16 of them yielded a well-resolved and unique trace in Sanger sequencing chromatograms. Thus, the remaining 8 sequences were submitted to the NGS approach together with the samples (*n* = 9) that gave inconclusive results in ELISA or in the PCR amplification steps, for a total of 17 samples. Among them, samples G011, G044 and G058 tested doubtful or negative in the PCR step and gave inconclusive results in the genotyping ELISA, and thus were further investigated using the NGS “blind” approach. All 17 samples gave a positive result in amplicon sequencing with an average depth of coverage that spanned from 2224 to 533,200×, having 14,430–2,583,436 aligned reads which represents ~100% amplicon coverage.

**Figure 2:**
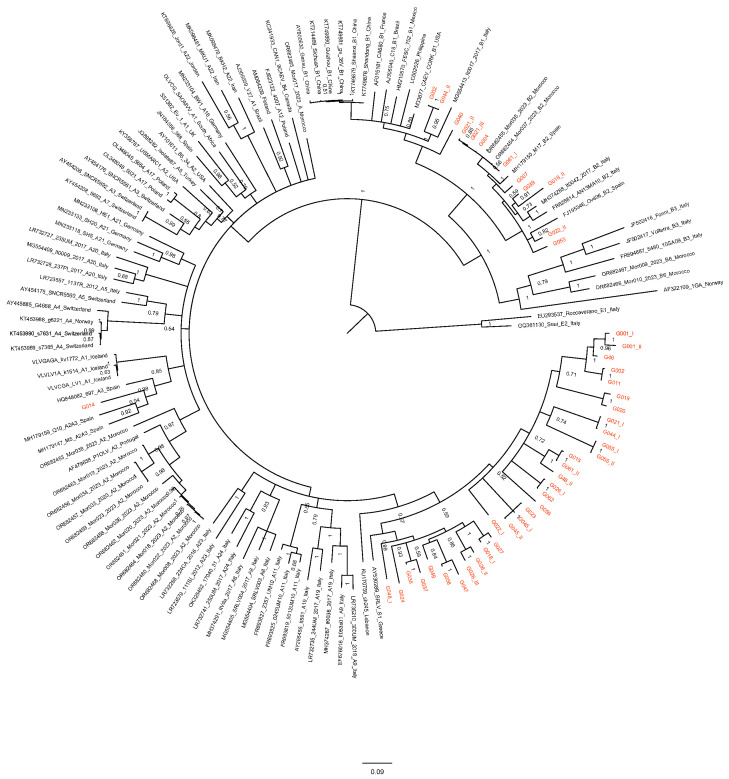
Phylogenetic tree based on the alignment of 780 bp of gag sequence of reference and newly characterized Greek strains. The accession number, name, subtype and country of detection are reported in the label for each reference sequence. Bootstrap values are shown above the respective branches. The bar indicates the amount of evolution along the horizontal branches in substitutions per site.

For 10 samples out of 17, the NGS protocol allowed us to identify more than one viral sequence into the sequencing reads belonging to each sample. In eight bulk milk samples, it was possible to retrieve two distinct viral sequences (named I and II in Figure 2 and Table 2 and Table 3), while in two samples, three different viral sequences were identified (named I, II and III in Figure 2 and Table 2 and Table 3). In more than one case, the sequences belonged to a distinct viral cluster (G018, G021, G022, G044 and G061). For example, the sample G018 returned two sequences: one belonging to the A genotype and the other one belonging to the B genotype, clustering together with the B2 strains. Samples G021 and G022 belonged to mixed farms while sample G044 belonged to a goat herd. Interestingly, G061 was a tissue sampled from the lung of a positive sheep with respiratory clinical signs, chosen as positive control. In this case, the NGS gave two distinct gag–pol viral sequences: one clustering with the B2 subtypes (34.05% of the aligned sequencing reads) and the other one with the new A Greek viral cluster (65.95% of the aligned sequencing reads), suggesting a possible co-infection.

Moreover, a great variability of each single sequence retrieved with the NGS approach was also recorded. The derived sequences contained multiple variation sites, from 8 to 32 with frequencies from 8 to 89%, likely representing the high mutation rate of these viruses in a population.

A total of 45 unique gag–pol sequences were retrieved and further investigated.

The phylogenetic analysis was based on the alignment of 780 nucleotides of the 45 sequences from Greece and 93 reference strains from groups A and B and rooted to group E.

The topology of the tree revealed the presence of both A and B genotypes (Figure 2 and Table 2). In particular, 12 sequences belonged to B subtypes while 33 belonged to A subtypes. Interestingly, 32 out of 33 clustered into a new viral cluster, showing a percentage of identities ranging from 87.2 to 93% with previously reported Greek sequences, from 83.3 to 86.6% with an A12 sequence from Poland, and from 84.2 to 86.4% with a previously reported sequence from Lebanon (Figure 2).

## 4. Discussion

Small ruminant lentiviruses are retroviruses that cause persistent infections in goats and sheep worldwide, resulting in significant economic losses, particularly in those countries in which small ruminant farming has an important social and financial role [34,35,36]. In the absence of an efficient vaccine or cure, control programs have been shown to be the only effective strategy to avoid the spread of the disease [17]. Control programs mainly rely on testing and removing positive animals. However, the genetic variability of SRLVs and the lack of gold-standard diagnostic tests able to identify all the possible genotypes and subtypes represent a challenge and limitation in these programs [15].

Several studies demonstrated that in those countries where no control measures are implemented, SRLVs are present with high prevalence and show a high number of different genotypes and subtypes [25,26,27,37,38,39,40].

Greece is characterized by one of the largest small ruminant populations in Europe [7], accounting for about 6.5% and 22% of sheep and goat numbers, respectively [41]. Thus, sheep and goat breeding is the most important animal farming industry in Greece in which SRLV infection can have a high economic impact. However, although the disease has been well-known since 1967 [8] when first described in East Friesian sheep imported from Germany, there is a lack of serological or molecular surveys in the country and scarce information about circulating strains is available to date.

In this study, we firstly investigated the herd-level prevalence of antibodies against SRLVs in bulk milk samples collected all over the country, including populations from the mainland and islands, for a total of 60 farms and 15 different municipalities. The overall herd-level prevalence of infection was around 52% (mainland and islands), confirming previous reports in Greece [8,42] and similar to that reported for Mediterranean milk breeds. However, in these previous studies [22,40], the within-herd seroprevalence was tested rather than herd-level prevalence. Nevertheless, our estimated seroprevalence should remain under debate, taking into consideration that the tested farms represent a limited percentage of the entire population. Regardless of the diseases under investigation, the serological screening of individual animals within the herd can be an invasive and expensive procedure that leads to finding alternative and cost-effective tools for assessing the herd infection status. In this context, the bulk milk collected in a dairy farm represents a valuable matrix for decreasing the cost of monitoring programs for infectious diseases [43,44]. Among the others, the use of bulk milk has the advantage of overcoming the risk of false-negative results in individual samples due to the decline of antibody secretion in milk over time [15]. However, some parameters must be taken into consideration for their application in disease detection and prevalence evaluation, such as viral dynamics, the concentration and persistence of pathogens or antibodies, and the expected herd prevalence and stage of lactation of individual animals [45].

The use of bulk milk samples for the initial estimation of SRLV seroprevalence in a flock has been suggested by many authors as a cost-effective and useful matrix [20,21,22,23,24,28,46,47]. The same ELISA test applied in this study has been already employed to identify and assess genotype E prevalence in 186 bulk milk samples in Sarda goats in 2010 in Italy [23].

In addition, our findings revealed higher seropositivity in the farms from the mainland, confirming the hypothesis of there being healthier populations in the islands, probably due to the implementation of strict controls during animal trading.

Interestingly, only 50% of the ELISA screening-positive samples were correctly serotyped (18/31). This result was partially expected, since bulk milk represents a difficult matrix in this context. The negative results may be due to the low prevalence of infection and the sensitivity of single-epitope-based immunoassays (as genotyping is) being lower compared with a multiepitope-based screening test [48,49]. Indeterminate results occurred when two different genotypes were present in the flock; on three occasions (samples 18, 21 and 22), two different genotypes were detected by NGS. However, a clear single genotype was detected by serotyping. This may be due to different prevalence of infection and/or higher antibody concentrations toward one genotype. A negative result or incorrect serotyping may also be related to mismatches in the sequences encoding the genotyping antigens used in the test [13,50] as previously reported. This latter hypothesis was partially confirmed in samples 14, 47 and 18, in which the sequence of the capsid epitope displayed one or more mismatches, especially at the C′-terminus, while mismatches at the N′ terminus appeared to be better tolerated. Overall, the data suggest that the serotyping of screening positive bulk milk samples may be of limited value compared to individual serum or milk samples.

Thus, the genetic characterization of SRLVs circulating in naturally infected goats and sheep was evaluated using a nested PCR [28] and NGS amplicon sequencing as previously reported [26].

With this approach, 45 unique sequences were obtained from 33 farms that gave positive or doubtful results in the serological and molecular survey.

Considering the high mutation rate of SRLVs and the bulk milk matrix used for proviral DNA extraction, it is not surprising that more than one sequence could be retrieved from one sample. Interestingly, in ten samples, a great variability was recorded, and in five of them two distinct A and B viral subtypes were found. The first belonged to the B2 subtype, firstly isolated in cases of arthritis in Spanish sheep [51,52] and now endemic in Mediterranean flocks without species restrictions [25,26,37,40,53,54,55,56]. However, the majority of sequences retrieved in the study were clustered in a new A subgroup closely related to previously reported Greek and Lebanese sequences [6,57]. These sequences showed a mean genetic distance from around 13.6 to 15.8% with the A12 Polish sequence and, based on the SRLV classification reported by Shah [3], can be tentatively assigned to a new A28 viral subtype. Moreover, B1 and A3 subtypes were also recorded.

Interestingly, a swab sample was collected as positive control from the lungs of a sheep with clinical signs of Maedi–Visna in a farm from Kilkis. This approach allowed us to identify a possible co-infection with both B2 and A28 subtypes, confirming the added value of NGS over Sanger sequencing in detecting viral variants in these highly variable genomes. The latter is linked with the investigated goat herds from Skopelos Island, where we saw the absence of SRLV-specific antibodies in all of them and doubtful PCR results, while NGS allowed the identification of A or B1 strains circulating among the tested animals.

It is noteworthy that, in light of the increasing number of viral subtypes that have been (and can be) discovered with high-throughput molecular techniques, the classification proposed by Shah at al. in 2004 may need revision in the near future.

This study, once again, highlighted the complex scenario behind SRLV diagnosis and the importance of constant monitoring of viral evolution, in particular in sequences encompassing diagnostically relevant immunodomains, in order to design specific diagnostic tests or adapt previously developed assays to support the implementation of effective control measures.

## 5. Conclusions

In conclusion, this study allowed us to confirm a high herd-level prevalence of SRLV infection in Greece, quite similar to that reported in other Mediterranean countries. Bulk milk testing represents a cost-effective and rapid approach for the wide-ranging screening of SRLV infection and for estimating disease prevalence at the herd level. The circulation of B1 and B2 subtypes was identified and new viral clusters referred to as genotype A were detected, suggesting that the heterogeneity of the MVV group may be even larger than expected.

## Figures and Tables

**Table 1:** Data about regions and numbers of farms tested.

Origin of Samples	Region	Farms Tested	Species	Nr of Animals
**Mainland**	Aitolokarnania	2	Goats	190
		3	Sheep	202
**Mainland**	Edessa	2	Goats	500
**Mainland**	Chalkidiki	6	Goats	3050
		2	Sheep	240
**Island**	Ios	10	Goats	706
		3	Sheep	175
		1	Mixed	80
**Island**	Karistos	1`	Sheep	70
		1	Mixed	354
**Island**	Kefalonia	4	Goats	450
		2	Sheep	230
**Mainland**	Kilkis	1	Goats	800
		2	Sheep	280
**Mainland**	Larisa	2	Goats	600
		1	Sheep	400
		1	Mixed	310
**Mainland**	Grevena	3	Mixed	1700
**Mainland**	Serres	1	Sheep	230
**Island**	Skopelos	5	Goats	510
**Mainland**	Thessaloniki	1	Goats	640
**Mainland**	Trikala	1	Sheep	250
**Mainland**	Xanthi	1	Sheep	200
**Island**	Zante	4	Sheep	410

**Table 2:** Serology and sequencing data results. Samples in which more than one sequence was retrieved from NGS are listed with Roman numerals (I, II, III) and ditto marks (“) that indicate the information above it is repeated.

Sample ID	Location	Species	ELISA Screening	ELISA Genotyping	PCR	Subtype
**G001_I**	Karistos	Sheep	Positive	Negative	Positive	A
**G001_II**	“	“	“	“	“	A
**G002**	Karistos	Mixed	Positive	B	Positive	A
**G004**	Ios	Sheep	Positive	B	Positive	B2
**G006**	Ios	Goat	Positive	Negative	Negative	-
**G007**	Ios	Goat	Positive	B	Positive	B2
**G011**	Ios	Goat	Positive	AB	Negative	A
**G014**	Ios	Goat	Positive	AB	Positive	A3
**G015**	Ios	Sheep	Positive	Indeterminate	Positive	A
**G018_I**	Zante	Sheep	Positive	A	Positive	A
**G018_II**	“	“	“	“	“	B2
**G019**	Zante	Sheep	Positive	Indeterminate	Positive	A
**G020**	Zante	Sheep	Positive	Indeterminate	Positive	A
**G021_I**	Grevena	Mixed	Positive	A	Positive	A
**G021_II**	“	“	“	“	“	B2
**G021_III**	“	“	“	“	“	B2
**G022_I**	Grevena	Mixed	Positive	A	Positive	A
**G022_II**	“	“	“	“	“	B2
**G023**	Grevena	Mixed	Positive	A	Positive	A
**G024**	Chalkidiki	Goat	Positive	Indeterminate	Positive	A
**G025**	Chalkidiki	Goat	Positive	Negative	Negative	-
**G026_I**	Chalkidiki	Goat	Positive	A	Positive	A
**G026_II**	“	“	“	“	“	A
**G026_III**	“	“	“	“	“	A
**G027**	Chalkidiki	Goat	Positive	A	Positive	A
**G032**	Kefalonia	Goat	Positive	B	Positive	B1
**G037**	Kilkis	Sheep	Positive	Indeterminate	Positive	A
**G038**	Kilkis	Sheep	Positive	Indeterminate	Positive	A
**G039**	Kilkis	Goat	Positive	AB	Positive	B2
**G040**	Xanthi	Sheep	Positive	AB	Positive	B1
**G041**	Edessa	Goat	Positive	Negative	Negative	-
**G043**	Chalkidiki	Sheep	Positive	A	Positive	-
**G044_I**	Skopelos	Goat	Negative	Negative	Doubt	A
**G044_II**	“	“	“	“	“	B1
**G045_I**	Skopelos	Goat	Negative	Negative	Doubt	A
**G045_II**	“	“	“	“	“	A
**G046**	Skopelos	Goat	Negative	Negative	Doubt	A
**G047**	Edessa	Goat	Doubt	Indeterminate	Positive	A
**G048_I**	Serres	Goat	Doubt	Negative	Positive	A
**G048_II**	“	“	“	“	“	A
**G049**	Larisa	Mixed	Positive		Positive	A
**G051**	Aitolokarnania	Goat	Doubt	Negative	Negative	-
**G052**	Larisa	Goat	Positive	A	-	-
**G053**	Larisa	Sheep	Positive	A	Positive	B2
**G055_I**	Aitolokarnania	Sheep	Positive	Negative	Positive	A
**G055_II**	“	“	“	“	“	A
**G056**	Aitolokarnania	Sheep	Positive	A	Positive	A
**G058**	Trikala	Sheep	Positive	Indeterminate	Doubt	A
**G061_I**	Kilkis	Sheep swab	Not applicable	Not applicable	Positive	B2
**G061_II**	“	“	“	“	“	A
**G062**	Messinia	Sheep swab	Not applicable	Not applicable	Positive	A

**Table 3:** Immunodominant capsid epitope sequence alignment. The newly characterized Greek sequences are aligned using reference strains belonging to different subtypes and countries. The subtype of each reference strain is indicated when available.

Strain	Subtype	Sequence
VLVLV1A_k1514_Iceland	A1	KLNEEAERWVRQNPPGP
AY101611_85-34_USA	A2	.................
AY530289_SRLV-S1_Greece		.................
G001_I		.................
G001_II		.................
G011		.................
G018_I		.................
G022_I		.................
G026_II		.................
G026_III		.................
G045_I		.................
G045_II		.................
G046		.................
G048_I		.................
G055		.................
G058		.................
G061_II		.................
G015		.................
G019		.................
G020		.................
G023		.................
G027		.................
G037		.................
G038		.................
MN233104_IIW1_Germany	A16	.........I.......
MG554409_It0009_2017_Italy	A20	.........I.......
AY530290_SRLV-S2_Greece	A	.........I.......
AF479638_P1OLV_Portugal	A2	..............GPN
AY530292_SRLV-S4_Greece	A	R................
G002		R................
G021_I		R................
G026_I		R................
G044_I		R................
G062		R................
G024		R................
G049		R................
AM084209_Finland		...D.............
G48_II		...D.............
G056		...D.............
AY445885_G4668_Switzerland	A4	..............A..
G047		..............Q..
AJ305039_V27_Brazil	A1	................Q
OR666874_RO46_Romania	A	................Q
G014		................Q
KC241933_CAN1_9CAEV_Canada	B4	................R
AF015181_CA680_France	B1	......D..R.N....G
M33677_CAEV_CORK_USA	B1	.........R.N...P.
G044_II		.........R.N...P.
G032		.........R.N...P.
G040		.........R.N...P.
LC002526_Philippine	B	......D..R.N...P.
FJ195346_Ov496_Spain	B2	.........R.N...PQ
G021_II		.........R.N...PQ
G021_III		.........R.N...PQ
G022_II		.........R.N...PQ
G061_I		.........R.N...PQ
G039		.........R.N...PQ
G053		.........R.N...PQ
G07		.........R.N...PQ
JF502416_Fonni_Italy	B3	.........R.N...PA
JF502417_Volterra_Italy	B3	.........R.N...PA
G018_II		.........KKN...PQ
G04		.......G.R.N...PQ
AF322109_1GA_Norway	C	.........R....QPA
EU293537_Roccaverano_Italy	E1	...K...T.M....QP.
GQ381130_Seui_Italy	E2	...K...T.M....QP.

## Data Availability

Sequencing data produced in this paper are available in GenBank with accession numbers from PP484895 to PP484939.

## Supplementary Materials

The following supporting information can be downloaded at: https://www.mdpi.com/article/10.3390/ani14071119/s1, Table S1: list of primers used in the study.
