# Supplementary material for: A Combined Approach for the Characterization of Small Ruminant Lentivirus Strains Circulating in the Islands and Mainland of Greece
Source: Animals (Basel). 2024 Apr 6;14(7):1119. doi: 10.3390/ani14071119 (PMC11010947; doi:10.3390/ani14071119)
Supplement: Supplementary file 1 [file animals-14-01119-s001.zip › animals-2938900-supplementary.pdf]

Supplementary Table S1

Primer name	Sequence	Position *
GAG 1st For	5′-TGGTGARKCTAGMTAGAGACATGG-3′	513 to 536
GAG F2 2nd For	5′-CAAACWGTRGCAATGCAGCATGG-3′	1010 to 1032
POL R1 1st Rev	5′-CATAGGRGGHGCGGACGGCASCA-3′	1830 to 1852
POL R24 2nd Rev	5′-GCGGACGGCASCACACG-3′	1826 to 1842

Table. Primers used in this study from Grego et al. 2007. Primer name, sequence and position related to CAEVGC reference sequence (GenBank Acc. Num. M33677.1) are provided.
